# Unclassified Inborn Errors of Immunity Patients without any Pathogenic Variant in Targeted Next-Generation Sequencing: Long-Term Follow-up and Whole Exome Sequencing Results

**DOI:** 10.1007/s10875-026-02007-z

**Published:** 2026-03-31

**Authors:** Ilke Bas, Ezgi Topyildiz, Emine Ulgen, Pinar Sahin, Gamze Tokgoz Erdis, Asude Durmaz, Neslihan Edeer Karaca, Guzide Aksu, Ayca Aykut, Necil Kutukculer

**Affiliations:** 1 Department of Pediatric Immunology, Faculty of Medicine, Ege University, Bornova, Izmir Türkiye; 2 Department of Medical Genetics, Faculty of Medicine, Ege University, Izmir, Türkiye

**Keywords:** Targeted next-generation sequencing, Whole exome sequencing, Unclassified primary antibody deficiency, Unclassified inborn errors of immunity

## Abstract

**Purpose:**

In this eight-year study, children with unclassified inborn errors of immunity (IEI) who have no pathogenic variants in targeted next-generation sequencing (tNGS) were re-evaluated by using singleton whole exome sequencing (sWES) and followed-up at least five years. They were also investigated if they had affected family members or not. In addition, all patients had detailed immunological and clinical work-up.

**Methods:**

In 2017, 108 children with probable diagnosis of IEI had tNGS and pathogenic variants were detected in 38 (35.1%) patients who were not included into the study. The rest 70 patients were continued to be immunologicaly followed-up (67.0 *±* 19.7 months) and 15 of them (21.4%) recovered spontaneously. Fifty-five patients were divided into two groups; (i) familial patients (n = 30); (ii) non-familial patients (n = 25). In all of them, sWES analysis was performed.

**Results:**

There were 19 affected fathers, 9 mothers and 16 siblings whereas consanguinity rate for parents was 8.5%. In sWES analysis, no genetic alterations were detected in 28 (50.9%) patients and 14 cases showed predefined pathogenic variants which are not associated with IEI. Probable IEI causing variants were found in 13 of 55 cases. However, after genotype/phenotype examinations and segregation analysis by sanger sequencing for their parents, we were able to define only three pathogenic variants in three cases, namely two for *IKBKB* gene and one for *PRKDC* in familial cases. Fifty-five cases were screened with sWES and only three pathogenic variants were revealed (3/55, 5.45%). When we add this result to tNGS finding (35.1%), all together 40.55% of unclassifed IEI cases could be defined by both of these genetic investigations.

**Conclusion:**

Even with NGS-based approaches improving IEI diagnoses, detection rates still remain below 50%. Whole genome sequencing in the near future would offer the advantage of much more comprehensive and accurate variant detections.

**Supplementary Information:**

The online version contains supplementary material available at 10.1007/s10875-026-02007-z.

## Introduction

Inborn errors of immunity (IEI) are monogenic disorders, and they are a heterogeneous group of diseases characterized by impaired immune response to pathogens. Inborn errors of immunity have also dysregulated immune function causing autoimmunity and inflammatory disorders [1]. Although more than 500 IEI conditions which were proven by genetic analysis were reported, there are still unknown numbers of undefined and unclassified immune deficiencies.

Almost 80% of symptomatic patients referred to Pediatric Immunology Departments receive clinical diagnoses consistent with European Society for Immunodeficiencies (ESID) diagnostic criteria. However, approximately 20% of them are followed without a definitive diagnosis and are referred to as “unclassified IEI” (unIEI) or “unclassified primary antibody deficiency” (unPAD) [2]. Recently, this entity entered the ESID definitions for clinical diagnosis (https://esid.org/Working-Parties/Registry-Working%20Party/Diagnosis-criteria).

Long-term follow-up of patients with unPAD or unIEI is essential to determine their clinical outcomes and the potential progression towards a definitive diagnosis [3]. During this follow-up, some young infants with initially unPAD diagnosis may normalize their immunoglobulin levels within four years of age showing the condition of transient hypogammaglobulinemia of infancy (THI); conversely, others may develop a persistent or very late recovered primary immune deficiency [4].

The increased availability of new technologies in genetic diagnostics has a very important role in the remarkable increase in identification of novel monogenic IEI and decrease in numbers of unPAD and unIEI over the past years. The integration of genetic diagnostics into clinical practice impacts patient care, enables early diagnosis, prevents time loss for exact diagnosis and facilitates personal treatment strategies [5].

Targeted next-generation sequencing (tNGS) encompasses a certain primer set amplifying a selected group of genes (e.g., 264 primary immunodeficiency genes) [6]. tNGS offers high-accuracy variant detection but does not provide new insights into the role of novel genes [6]. On the other hand, whole exome sequencing (WES) enables the analysis of almost all genomic protein coding regions, which only represent about 1% of the entire genom but account for 85% of disease-causing pathogenic variants and can be performed on the proband only (singleton; sWES) or with additional samples, often including both biological parents with the proband (trio; tWES). Because of diagnostic advantage most of the genetic laboratories have switched to WES [7, 8].

In our previous study with a very large cohort of common variable immune deficiency (CVID) patients, in 35% of patients disease-causing pathogenic variants were detected by using tNGS [9]. The CVID patients without previously identified genetic variants were re-evaluated using sWES, which elucidated the genetic basis in an additional 5% of cases [9]. In the present study, we enrolled patients with IEI who had no detectable pathogenic variants in tNGS and re-evaluated them using sWES to investigate their genetic and molecular etiology. In addition, after extended follow-up periods, the unrevealed cases were also investigated if they had affected family members or not. Besides genetic analysis, all the patients had detailed immunological and clinical work-up for genotype/phenotype correlations.

## Patients and Methods

**Fig. 1:**
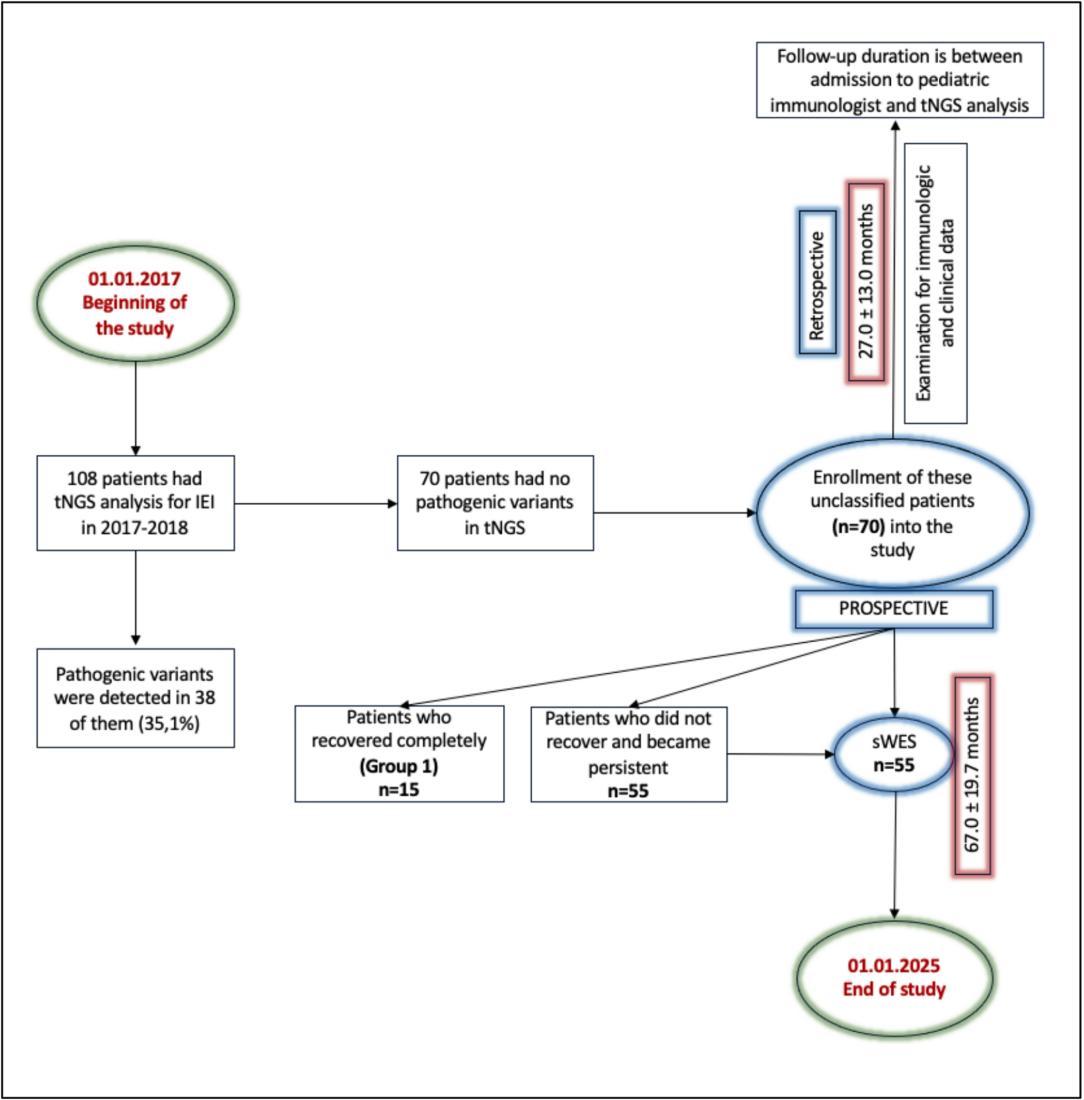
Study protocol

The study protocol is in Figure-1 and inclusion and exclusion criteria for the patients is below.

### Inclusion Criteria

(i) unPAD patients (with a marked decrease in at least one of total IgG, IgG1, IgG2, IgG3, IgA or IgM levels and/or failure of specific IgG antibody response to vaccines, plus one of the followings: recurrent bacterial infections or autoimmunity or lymphoproliferation or affected family member) (ii) other unIEI patients except unPAD cases without any pathogenic variant in tNGS analysis plus, (a) at least one major infection (b) at least one manifestation of immune dysregulation, (c) failure to thrive, (d) affected family member, (e) at least one numeric or functional abnormal finding upon immunological investigation and exclusion of secondary causes for immunological abnormalities, iii) Enrollment of these patients into the study after detecting no pathogenic variant in tNGS.

The other inclusion criterias were as follows: (i) Patients were required to be older than four years of age at the time of the tNGS analysis.; (ii) the included patients have to be followed-up at least five years with 6 months’ interval after enrollment; (iii) for unPAD patient, serum immunoglobulin levels have to be lower at least two standard deviations than normal levels for age-matched healthy controls.

### Exclusion Criteria

(i) THI patients (their immunoglobulins normalized within four years of age), (ii) selective IgA deficiency patients, common variable immunodeficiency patients with defined pathogenetic variants, X-linked agammaglobulinemia patients, class-switch recombination defects because of *CD40*,* CD40L* and *AID* defects, (iii) Genetic syndromes and chromosomal abnormalities with hypogammaglobulinemia and/or cellular immunity defects.

For the period before enrollment, clinical and immunological data of all patients were examined retrospectively. After tNGS analysis and enrollment, all work-up was performed prospectively for the following criteria:

1. Duration of follow-up both before and after tNGS and total duration
2. Type and localization of infections
3. Family history (if there is somebody in the family who have abnormal immunologic findings) and the presence of consanguinity
4. Age at admission and ages when tNGS and sWES was performed (month)
5. Complete blood count to examine if there is leukopenia and/or neutropenia
6. Serum concentrations of IgG, IgM, IgA at admission and during follow-up and their comparison with age-related normals.
7. Percentages and absolute counts of lymphocyte subsets
8. Serum IgG antibody responses against routine vaccine antigens
9. Segregation analysis by sanger sequencing for the parents when possible pathogenic variant was detected in sWES.
10.  Immunologic work-up of parents and siblings.
11.  Comparison of familial case group (Group 2) and non-familial case group (Group 3). A “familial case” was defined as a patient with at least one affected-first degree relatives [10].

Immunologic work-up of these patients included serum immunoglobulins by nephelometry (Dade Behring BN2 Nephelometer, Germany), lymphocyte penotyping by flow cytometry (Beckman Coulter Gallios flow cytometer and BC Kaluza Analysis Software) and specific IgG antibody response against routine vaccine antigens namely hepatitis B, hepatitis A, measles, rubella, varicella zoster and tetanus by ELISA technique.

The targeted next-generation sequencing analysis panel included 264 genes (Supplement).

### DNA Extraction

Genomic DNA was isolated from whole blood samples using the QIAamp DNA Blood Mini Kit (Qiagen, Hilden, Germany), following the manufacturer’s protocol. The concentration of the extracted DNA was determined using the Qubit™ dsDNA HS Assay Kit on a Qubit 2.0 Fluorometer (Thermo Fisher Scientific), in accordance with the provided instructions.

### Targeted Next-generation Sequencing

Library preparation was performed using the Ion Chef System (Thermo Fisher Scientific, San Francisco, CA, USA) following the manufacturer’s instructions. Barcoded libraries were constructed using 10 ng of DNA per sample with the Ion AmpliSeq Chef Solutions kit and the Ion AmpliSeq™ Primary Immune Deficiency Research Panel v2 (Thermo Fisher Scientific), which comprises 5241 amplicons across 264 genes. The prepared libraries underwent clonal amplification onto ion sphere particles (ISPs) via emulsion PCR using the Ion Chef System, in accordance with the provided protocols. Enriched ISPs were then loaded onto Ion 530 chips, each capable of processing 16 samples per sequencing run. Sequencing was carried out on an Ion S5 Sequencer using the Ion 530 Chip and the Ion 530 kit–Chef Kit (Thermo Fisher Scientific). Sequence alignment to the hg19 reference genome and base calling were performed using Torrent Suite software.

### Whole Exome Sequencing

Library preparation was performed using the KAPA HyperExome/HyperPlus Kit (Roche, Basel, Switzerland), and sequencing was carried out on the DNBSEQ-G400 platform (MGI Tech, Shenzhen, China) with a minimum coverage depth of 100×.

Raw sequencing data were processed using the Genomize SEQ™ platform for quality control, alignment to the reference genome (GRCh38), variant calling, and annotation [11]. Variants were filtered to retain nonsynonymous changes with minor allele frequency (MAF) < 0.01 in the Genome Aggregation Database (gnomAD) [12], the Exome Aggregation Consortium (ExAC) [13] and 1000 Genomes (The 1000 Genomes Project Consortium) (2015) [14] and < 0.05 in our in-house database. Intronic variants within ± 10 bp of exon–intron junctions and potential non-canonical splice site variants were also considered. Copy number variations (CNVs) and mitochondrial variants were not included due to limitations of the exome capture design.

Variant prioritization incorporated inheritance mode, clinical phenotype, database entries (HGMD) (ClinVar) [15, 16] and Franklin [17] and Varsome [18] platforms were used to integrate clinical and computational data. Finally, variant classification followed the American College of Medical Genetics and Genomics (ACMG) - Association for Molecular Pathology (AMP) guidelines to identify likely pathogenic and pathogenic variants relevant to the patient phenotype [19].

After library preparation, sequencing was conducted on the DNBSEQ-G400 system. The sequencing yielded an average read depth of > 140× across 96% of the targeted exonic regions, providing robust and reliable data for downstream analysis.

### Variant Interpretation

The clinical significance of novel genetic variants was assessed using the standards and guidelines established by the ACMG in collaboration with the AMP. Minor allele frequencies were evaluated by referencing publicly available population databases, including the NCBI dbSNP (build 141), the 1000 Genomes Project, the ExAC, and the gnomAD. These resources provided valuable insights into the population prevalence of the identified variants.

Variant-specific disease associations were further investigated using databases such as ClinVar and the Online Mendelian Inheritance in Man (OMIM), which offer curated information on genetic variants and their links to clinical phenotypes. To predict the potential impact of novel variants on protein structure and function, a variety of in silico tools were employed, including GERPP, PolyPhen-2, and SIFT. These tools helped to classify the variants’ potential effects on protein stability, function, and pathogenicity.

Following ACMG guidelines, the identified variants were categorized into pathogenic, likely pathogenic, or variants of uncertain significance (VUS). For novel variants, detailed analyses were conducted to evaluate their pathogenicity, mode of inheritance, and associated clinical phenotypes. Candidate pathogenic variants detected through tNGS were subsequently validated using Sanger sequencing on an ABI PRISM 3500 DNA Analyzer (Applied Biosystems). Sanger sequencing was utilized to validate ambiguous variants identified in the tNGS data, thereby ensuring the accuracy and reliability of the results.

After variant validation, segregation analysis was performed to examine inheritance patterns within affected families. These mutations were systematically classified as pathogenic, likely pathogenic, or VUS in line with ACMG criteria, providing a comprehensive framework for understanding their clinical relevance.

### Statistical Analyses

Data were evaluated using the Statistical Package for Social Sciences 25.0 (SPSS for Windows 25.0, Inc, Chicago, IL, USA) and by analyzing descriptive statistics (mean, standard deviation, median, minimum, and maximum). Categorical variables in dual groups were compared using Chi-square test. A p-value of < 0.05 was considered significant for all analyses.

## Results

In 2017–2018 years, 108 children with probable diagnosis of IEI had tNGS analysis in Ege University, Department of Medical Genetics. A novel or previously known pathogenic variants were detected in thirty-eight (35.1%) of them namely *BTK* (n:4), *TACI* (*TNFRSF13B*) (n:14), *STAT3* (n:1), *ATM* (n:2), *LRBA* (n:2), *CD40L* (n:1), *TRNT-1* (n:1), *RAG-2* (n:2), *BLNK* (n:1), *AIRE* (n:1), *Phoxp3* (n:2), *TTC37* (n:1), *PNP* (n:1), *IFNGR1* (n:1), *CGD-CyBA* (n:1), *PLCƔ2* (n:3). These patients had exact diagnosis and they were not included into the study.

In tNGS analysis, 70 patients did not have any pathogenic variants and these patients were accepted as undefined/unclassified IEI and they were enrolled into the study. The mean age of included patients (n = 70) was 45.0 *±* 20.6 months at admission and 72.0 *±* 25.1 months at enrollment (just after tNGS analysis) (Table-1).

**Table 1:** Demographic, laboratory and clinical findings of the study group

	*n*/*n* (%)	
Consanguinity rate	6/70 (8.5)	
Total number of all screened father-mother-siblings	120	
Familial case	33/70 (47.1%)	
Affected father	19/33 (57.5%)	
Affected mother	9/33 (27.2%)	
Affected sibling/siblings	16/33 (48.4%)	
Patients receiving intravenous immun globulin (IVIG) treatment before enrollment	56/70 (80.0%)	
Abnormalities in complete blood count		
Patients with leucocytopenia	4/70 (5.7%)	
Patients with neutropenia	6/70 (8.5%)	
Antigen-specific responses to vaccination at admission		
Positive anti-HBs antibodies	27/32 (84.3%)	
Positive anti-HAV IgG antibodies	17/23 (73.9%)	
Positive anti-measles IgG antibodies	8/12 (66.6%)	
Positive anti-rubella IgG antibodies	12/13 (92.3%)	
Positive anti-mumps IgG antibodies	8/13 (61.5%)	
Positive anti-tetanus IgG antibodies	8/14 (57.1%)	
Symptoms at admission and follow-up		
Upper respiratory tract infections	48/70 (68.5%)	
Otitis media	14/70 (20.0%)	
Sinusitis	1/70 (1.4%)	
Bronchiolitis	26/70 (37.1%)	
Bronchopneumonia	16/70 (22.8%)	
Asthma bronchiale	2/70 (2.8%)	
Acute gastroenteritis	15/70 (21.4%)	
Aftous stomatitis	3/70 (4.2%)	
Urinary tract infection	7/70 (10.0%)	
Skin disorders	7/70 (10.0%)	
Febrile convulsion	1/70 (1.4%)	
	**Mean** **±** **SD**	**Median (min-max)**
Age at hospital admission (months)	45.0 *±* 20.6	32 (14–170)
Age at study enrollment (months)	72.0 *±* 25.1	60 (45–174)
Age at WES analysis (months)	120.2 *±* 37.2	90 (65–208)
Follow-up time before enrollment (months) (retrospective)	27.0 *±* 13.0	20 (3-130)
Follow-up time after enrollment until WES analysis (months) (prospective)	43.4 *±* 20.9	4 (4–86)
Total follow-up time after enrollment until the end of the study (months) (prospective)	67.0 *±* 19.7	68 (14–96)

The follow-up time of study group before enrollment was 27.0 *±* 13.0 months. Immunologic and clinical data of this period were examined retrospectively from their medical records (Figure-1). After tNGS and negative result, they were continued to be immunologicaly followed-up (67.0 *±* 19.7 months) and 15 of them (21.4%) recovered completely (Group1) (Figure-1). After their spontaneous recovery, these patients were diagnosed as Hyper-IgM type IV (n = 7), partial IgA deficiency (n = 4), IgG2-3 deficiency (n = 3) and IgM deficiency (n = 1).

The rest of the patients (n = 55, 78.6%) were divided into two groups; (i) patients who did not recover and have family members with immune deficiences (familial cases) (n = 30) (Group 2); (ii) patients who did not recover and do not have family members with immune deficiences (n = 25) (non-familial cases) (Group 3). All the patients in these two groups had sWES analysis in the second prospective follow-up (Figure-1). The mean age of the patients who received sWES analysis was 120.2 *±* 37.2 months. Forty-seven of these unIEI patients were unPAD cases (85.4%) and eight were T cell and/or T/B cell defects (14.6%).

Upper respiratory tract infections, bronchiolitis, bronchopneumonia, and otitis media were the most common infections observed in study group patients (Table-1). 80% (56/70) of cases were receiving intravenous immunoglobulin (IVIG) therapy in order to reduce the rate of severe infections. As an additional risk factor for these infections, leucocytopenia (5.7%) and neutropenia (8.5%) were not highly observed in study cases (Table-1).

Specific IgG antibodies against some routine vaccines were determined at admission before any IVIG replacement. Highest antibody response was obtained against hepatitis B surface antigen (84.3%) while it was lowest for tetanus antibodies (57.1%) (Table-1).

Before tNGS and enrollment into the study, IgG values in study group patients were examined retrospectively by dividing them in three age groups; 0–2 years, 2–5 years and older than five years and they were all lower than age-related normal values (Table 2) [20]. In addition, mean percentages and absolute counts of T cells, T cell subsets, B cells and natural killer cells were all normal when they were calculated as a whole group (Table 3). However, when they were investigated individually, the minumum T cell level was 42% (408 cells/mm^3^) in a patient whereas minimum B cell level was 3.1% (29 cells/mm^3^) in another patient (Table 3). There were T and B cell alterations in eight patients.

**Table 2:** Serum immunoglobulin levels of the patients in study group

	Age: 0–2 years	Age:2–5 years	Age: >5 years
Normal IgG values*	682.3 *±* 195.6	850.5 *±* 182.5	1043.7 *±* 226.5
Before tNGS	n = 28	n = 20	n = 20
IgG (mg/dl)			
Mean *±* SD	358.0 *±* 171.0	445.0 *±* 95.5	538.2 *±* 192.8
(median) (min-max)	(340) (87–764)	(438) (292–633)	(552) (219–1110)
IgA (mg/dl)			
Mean *±* SD	24.2 *±* 24.1	45.9 *±* 19.7	56.5 *±* 32.7
(median) (min-max)	(19) (5-131)	(42) (17–87)	(52) (7-127)
IgM (mg/dl)			
Mean *±* SD	61.6 *±* 35.4	67.5 *±* 29.0	66.8 *±* 29.9
(median) (min-max)	(51) (8-140)	(65) (17–121)	(67) (18–143)
After tNGS			n = 70
IgG (mg/dl)			
Mean *±* SD	NA	NA	607.2 *±* 144.3
(median) (min-max)			589 (347–1009)
IgA (mg/dl)			
Mean *±* SD	NA	NA	69.0 *±* 39.2
(median) (min-max)			60 (6.5–192)
IgM (mg/dl)			
Mean *±* SD	NA	NA	73.9 *±* 36.8
(median) (min-max)			62 (18–174)

*Normal values, see in reference 10.

NA: not applicable

**Table 3:** Percentages and absolute counts of lymphocyte subsets of the patients in the study group

All patients before tNGS *n*:70	%		/mm3	
	mean *±* SD	median (min-max)	mean *±* SD	median (min-max)
CD3 + T lymphocyte	67.8 *±* 8.7	69 (42–90)	2987 *±* 1587	2700 (408–7493)
CD19 + B lymphocyte	20.1 *±* 7.6	19 (3.1–49)	927 *±* 655	727 (29-3014)
CD4 + T helper	40.7 *±* 8.6	40 (24.5–60)	1814 *±* 1049	1599 (238–4729)
CD8 + T cytotoxic	22.9 *±* 5.8	22 (9.7–38)	972.6 *±* 518.4	857.7 (134–2678)
CD16 + 56+ Natural killer	9.3 *±* 4.9	9 (0.6–25)	407.3 *±* 332.9	293 (13-1765)

We have screened immunological findings of first-degree relatives (father, mother, and siblings) (n = 120) of all study group. There were 33 “familial cases” (33/70) (47.1%). Three familial cases with affected siblings recovered spontaneously and the rest of them did not (n = 30) (Group 2). There were 19 affected fathers, nine mothers and 16 siblings (Table-1). Some patients had only one affected family member and some of them had more than one. In addition, consanguinity rate in parents was 8.5% in study group.

**Fig. 2:**
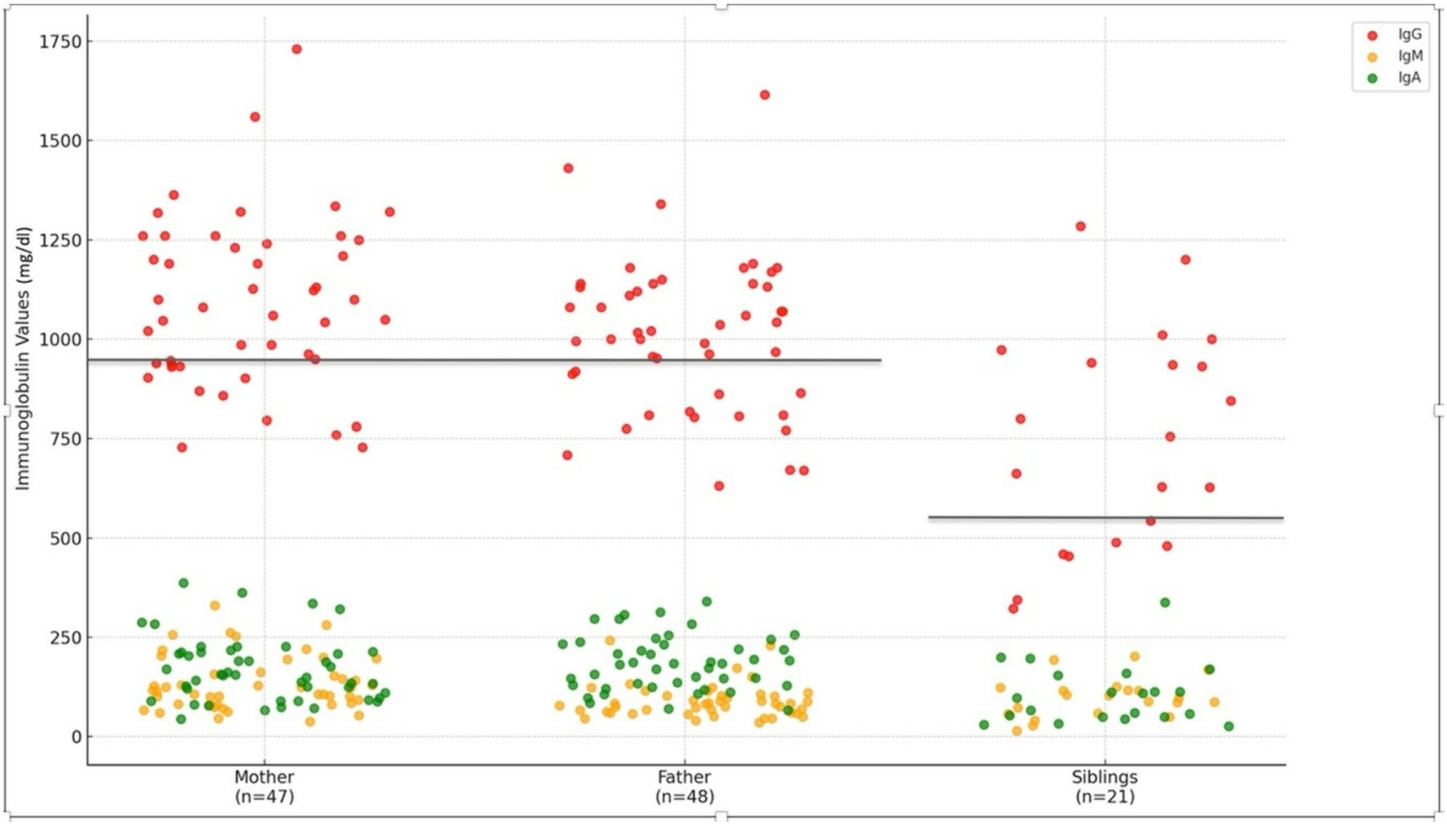
The distribution of immunoglobulin values in the family members all the patients

In Figure 2, the distribution of immunoglobulin levels of the family members was shown. IgG is accepted to be low in fathers and mothers if it is less than 950 mg/dl. IgG levels lower than 500 mg/dl is also considered low in children [20]. Figure 2 shows the decreased immunoglobulin values in family members.

**Fig. 3:**
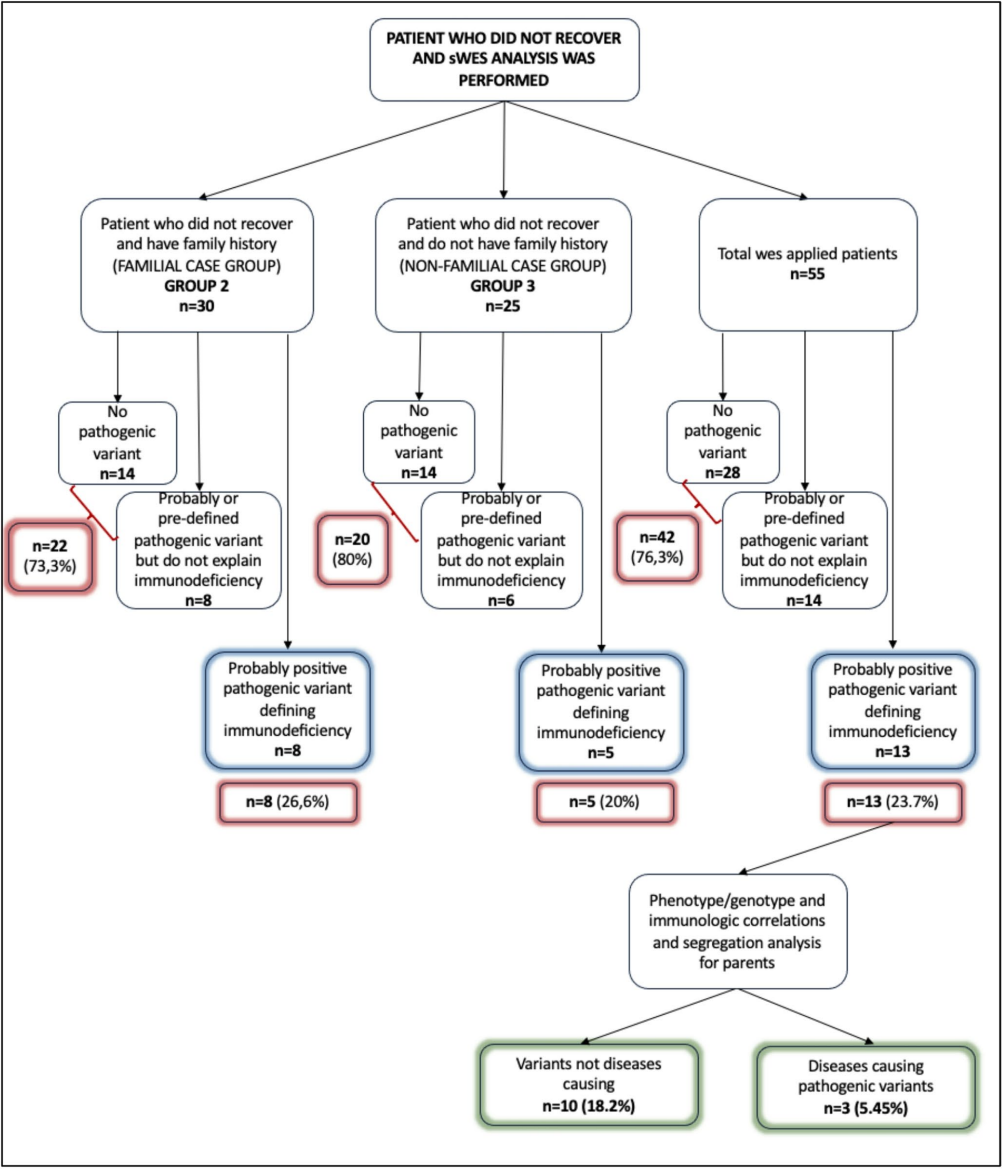
Genetic investigations of all patients who did not recover spontaneously

In sWES analysis, no genetic alterations were detected in 28 patients (28/55, 50.9%), 14 cases both in Group 2 (14/30, 46.6%) and Group 3 (14/25, 56%) (*p* > 0.05) (Fig. 3).

On the other hand, eight cases in Group 2 (8/30, 26.6%) and six cases in Group 3 (6/25, 24%) showed predefined pathogenic variants which are not associated with IEI (Fig. 3). These genetic variants were listed in Table-4. There were several genes such as *ADA*, *RAG2* and *TLR3* which may be immunologic disease causing, but their inheritance type or being in a VUS or benign characteristics did not lead us to explain the pathogenesis of immunodeficiency. On the other hand, two children learned that they are carrier for cystic fibrosis and one patient who was hospitalized before with hemolytic uremic syndrome diagnosis became clear for his genetic predisposition (Table-4).

**Table 4:** Detected genetic variants that do not explain immunodeficiency in 14 cases from both groups

Patient	Gene	Phenotype	Inheritance	Zygosity	Variant	ACMG	Clinic and laboratory association
**1) E.Y.Y** **Group 2**	FILAGGRIN; *FLG*	Susceptibility to atopic dermatitis	AD	Heterozygous	c.4297 G > T (p.Glu1433*)	Likely Pathogenic	No matching clinical signs
		Ichthyosis vulgaris	AD, AR				
**2) K.U.O** **Group 2**	ADENOSINE DEAMINASE; *ADA*	Severe combined immunodeficiency due to ADA deficiency	AR	Heterozygous	c.302G > A (p.Arg101Gin)	Pathogenic	Carrier for ADA SCID, normal lymphocyte subsets
**3) A.E** **Group 2**	CYSTIC FIBROSIS TRANSMEMBRANE CONDUCTANCE REGULATOR; *CFTR*	Cystic fibrosis	AR	Heterozygous	c.1521–1523 del (p.Phe508del)	Pathogenic	Carrier for cystic fibrosis
**4) E.K** **Group 2**	UDP-GLYCOSYL-TRANSFERASE 1 FAMILY, POLYPEPTIDE A1; *UGT1A1*	Crigler-Najjar syndrome, type I and II	AR	Heterozygous	c.211G > A (p. p.Gly71Arg) Rs:4,148,323	Likely Pathogenic	Carrier
	LAMININ, ALPHA-4; *LAMA4*	Cardiomyopathy, dilated	AD	Heterozygous	c.1261G > A(p.Val421Met)	VUS(PM2, BP4)	Normal cardiac examination
**5) Y.A** **Group 2**	LYSOSOMAL TRAFFICKING REGULATOR; *LYST*	Chediak-Higashi syndrome	AR	Heterozygous	c.3739G > C (p.Gly1247Arg)	VUS (PM2, BP4)	No matching clinical and laboratory signs with these variants
	RECOMBINATION-ACTIVATING GENE 2; *RAG2*	Severe combined immunodeficiency	AR	Heterozygous	c.1390 C > T(p.Arg464Cys)	VUS(PM2, PM1, PP2)	
	CYTOTOXIC T-LYMPHOCYTE ASSOCIATED 4; *CTLA4*	Immune dysregulation with autoimmunity, immunodeficiency, and lymphoproliferation	AD	Heterozygous	c.326G > A(p.Gly109Glu)	VUS(PM2, PP2, BP4)	
**6) A.L** **Group 2**	LIPID-BINDING CHAPERONE; *UNC119*	Immunodeficiency 13	AD	Heterozygous	c.593 C > T (p.Pro198Leu)	VUS (PM2)	Normal lymphocyte subsets, no CD4 + lymphopenia, CVID phenotype
**7) Ç.K** **Group 2**	CD46 ANTIGEN; *CD46*	Hemolytic uremic syndrome, atypical,	AR, AD	Heterozygous	c.828G > T (p.Trp276Cys)	VUS (PM2, PP3)	This patient had HUS + CVID clinic + On fSCIG therapy
**8) A.I** **Group 2**	PROTOPORPHYRINOGEN OXIDASE; *PPOX*	Variegate porphyria, childhood-onset	AR	Homozygous	c.1243 C > T (p.His415Tyr)	VUS (PM2, PP2)	No matching sign
**9) T.G** **Group 3**	TOLL-LIKE RECEPTOR 3; *TLR3*	Immunodeficiency, susceptibility to viral infections	AR, AD	Heterozygous	c.442–2 A > G	VUS (PM2)	No associated sign Patient had hypogammaglobulinemia and bacterial infections
**10) S.Ç** **Group 3**	LAMININ, BETA-3; *LAMB3*	Amelogenesis imperfecta, type IA	AD	Heterozygous	c.1117 C > T (p.Gln373Ter)	Pathogenic	No associated clinical signs for both genetic variants
	TSC COMPLEX SUBUNIT 2; *TSC2*	Tuberous sclerosis-2	AD	Heterozygous	c.-30 + 2T > C	﻿VUS(PM2, PVS1, BP6)	
**11) K.A** **Group 3**	DYNEIN, AXONEMAL, HEAVY CHAIN 11; *DNAH11*	Ciliary dyskinesia, primary	AR	Heterozygous	c.10,055 C > T (p.T3352M)	VUS (PM2)	No associated clinical signs for both genetic variants
	SOLUTE CARRIER FAMILY 25 MEMBER 10; *SLC25A10*	Mitochondrial DNA depletion syndrome 19	AR	Heterozygous	c.1129 C > T(p.R377*)	VUS(PM2)	
**12) A.Ş** **Group 3**	CYSTIC FIBROSIS TRANSMEMBRANE CONDUCTANCE REGULATOR; *CFTR*	Cystic fibrosis	AR	Heterozygous	c.220 C > T (p.R74W)	VUS (PM1, PP2, PM5, BS1, BS2, PP5)	Carrier for both diseases
**13) Ö.E.A** **Group 3**	PHOSPHOGLYCERATE KINASE 1; *PGK1*	Phosphoglycerate kinase 1 deficiency	XLR	Hemizygous	c.758T > C (p.Ile253Thr)	Likely Pathogenic	No associated sign
**14) Ö.S.A** **Group 3**	CALCIUM CHANNEL, VOLTAGE-DEPENDENT, ALPHA-1E SUBUNIT; *CACNA1E*	Developmental and epileptic encephalopathy	AD	Heterozygous	c.1133G > A (p.Arg378His)	VUS (PM2, PP3, PP2)	No associated sign

In sWES studies, probable disease-causing variants defining immunodeficiencies were found in 13 of 55 cases without any genetic findings in tNGS analysis. These variants were Inhibitor of nuclear factor kappa-B kinase, subunit beta *(IKBKB)* (n = 3), *Sect. 23 B*, coat complex II component, Bromodomain and WD repeat domain-containing protein 1 *(BRWD1*), Elastase, neutrophil-expressed *(ELANE)*, Interferon regulatory factor 5 *(IRF5)*, Keratin 5, type II (*KRT5*), Protein kinase, DNA-activated, catalytic subunit (*PRKDC*), Protein tyrosine kinase SYK (n = 2), *SPI1 protooncogene* and in Chr22:23180706–24,526,062 region duplication for 1,345,357 (Table-5). In order to understand whether these variants were disease causing or not, these 13 patients were examined for genotype/phenotype characteristics, and they had segregation analysis by sanger sequencing for their parents. After these investigations, we were able to define only three pathogenic variants in three cases, namely two for *IKBKB* gene and one for *PRKDC* (Table-5).

Two groups with (n = 30) or without (n = 25) familial cases were compared for detected disease-causing variants. Although, familial case group (Group 2) had much more positive detections (8/30-26.6% vs. 5/25 − 20%) than Group 3, it was not statistically significant (*p* > 0.05). All three disease causing variants were found in familial cases group.

Fifty-five cases were screened with sWES and only three pathogenic variants were found 3/55, 5.45%). When we add this result to tNGS finding (35.1%), altogether 40.55% of unclassifed IEI cases could be defined by means of genetic investigations. The benefit of additional sWES analysis for tNGS analyzed negative patients was found to be limited.

## Discussion

This study has some unique aspects and differences from other studies and also some novelties such as follows; (a) it has a study protocol that was performed for the first time with both tNGS and sWES analysis and with an eight-year retrospective and prospective follow-up of IEI patients, (b) Fifty-five patients has been examined not only with tNGS or not only with sWES as it was studied in previous studies, they were examined with both two different methods for the first time, (c) To our knowledge, different from all other studies, study cohort was not ordinary immune deficiency patients; a cohort of 55 unclassified/undefined IEI were followed-up and genetically examined, (d) it is also a unique study comparing the rate of detecting pathogenic variants between familial and non-familial cases.

The advent of NGS in 2010 has fascilitated the diagnosis of monogenic inborn errors of immunity, including primary immunodeficiencies [21]. In the retrospective part of this study and by using tNGS with 264 genes (suppl.), we detected disease causing variants in 35.1% of 108 children with IEI. Our present study group was heterogenous with very different IEI patients. However, in our previous study with only CVID patients, our detected disease-causing variants rate by tNGS was the same, 35.0% (8). In India, Rawat A et al. [21] used a targeted, customized gene panel of 44 genes known to result in IEI patients. Of 121 included patients, pathogenic variants were identified in 77 patients (63%) [22]. In another study by Grossi A et al. [23], tNGS gene panels composed of 58, 146 and 312 genes were used for 272 probable IEI patients. In a total of 37 patients (13.6%), disease causing pathogenic variants were detected. 

Fifty-five patients who did not recover spontaneously had second genetic testing (sWES) in addition to tNGS. In 28 of them (50.9%), there wasn’t any pathogenic variant and 14 patients (25.3%) showed pre-defined pathogenic variants not related with IEI (Fig. 2; Table-4). Some of these fourteen cases have learned their carrier status for autosomal recessive inherited diseases such as cystic fibrosis, Crigler-Najjar Syndrome (Table-4). In total 76.3% of twice genetically analyzed patients, no exact or probable disease-causing variants for IEI was found (Fig. 3).

In the second genetic analysis by using sWES, 23.7% of patients (13/55) showed “probable” disease-causing variants for IEI. However, after phenotype/genotype and immunologic correlations and also after segregation analysis of the parents by sanger sequencing only three variants were found to be disease causing, namely two for *IKBKB* gene and one for *PRKDC*. One more variant for *IKBKB* gene was accepted as “VUS” or “benign”, because same variant was detected in asymptomatic father with normal immunoglobulin values (Case 1, Table-5).

**Table 5:** Detected genetic variants probably explaining immunodeficiency in 13 cases from both groups

Patient	Gene	Phenotype	Inheritance	Zygosity	Variant	ACMG
**1) S.A** **Group 2**	INHIBITOR OF NUCLEAR FACTOR KAPPA-B KINASE, SUBUNIT BETA; *IKBKB*	Immunodeficiency	AR, AD	Heterozygous	c.2066 C > T (p.Ser689Phe)	VUS (PM2)
**2) E.C** **Group 2**	Section 23 HOMOLOG B, COAT COMPLEX II COMPONENT; *SEC23B*	Cowden syndrome 7 (rarely immunodeficiency)	AD	Heterozygous	c.325G > A (p.Glu109lys)	Pathogenic
**3) C.C** **Group 2**	INHIBITOR OF NUCLEAR FACTOR KAPPA-B KINASE, SUBUNIT BETA; *IKBKB*	Immunodeficiency 15 A	AD	Heterozygous	c.1079 C > T (p.Ala360Val)	VUS (PM2,BP4)
**4) E.B** **Group 2**	BROMODOMAIN AND WD REPEAT DOMAIN-CONTAINING PROTEIN 1; *BRWD1*	Hypogammaglobinemia (PMID:30250168) (ACMG)	AD	Heterozygous	c.4391 C > G (p.ser1464cys)	VUS (PM2)
**5) D.M.** **Group 2**	Cytoband start q11.22 (gain) Cytoband ebd q11.23 size 1929 (microarray)	In Chr22:23180706–24,526,062 region duplication for 1,345,357 Is this duplication cause hypogammaglobulinemia	-		ArrGRCH38 22q11 22q11.23 (22.508.57 9_24537.101)	VUS
**6) M.K.G** **Group 2**	INHIBITOR OF NUCLEAR FACTOR KAPPA-B KINASE, SUBUNIT BETA; *IKBKB*	Immunodeficiency 15 A	AD	Heterozygous	c.1079 C > T (p.Ala360Val)	VUS (PM2,BP4)
**7) Z.S** **Group 2**	ELASTASE, NEUTROPHIL EXPRESSED; *ELANE*	Neutropenia, severe congenital	AD	Heterozygous	c.68-13G > A	VUS (PM2,BS6)
**8) A.A** **Group 3**	KERATIN 5, TYPE II; *KRT5*	Epidermolysis bullosa simplex (hypogamma in some cases)	AD	Heterozygous	c.1675 C > T (p.Arg559Ter)	Likely Pathogenic
**9) D.Y** **Group 3**	INTERFERON REGULATORY FACTOR 5; *IRF5*	Inflammatory bowel disease	-	Heterozygous	c.501_530del (p.Arg175Leu 184del)	VUS (PM2)
**10) G.K** **Group 2**	PROTEIN KINASE, DNA-ACTIVATED, CATALYTIC SUBUNIT; *PRKDC*	Immunodeficiency	AR	Homozygous	c.10146T > C (p.Phe3382Leu)	VUS (PM2)
**11)İ. S.** **Group 3**	PROTEIN-TYROSINE KINASE SYK; *SYK*	Immunodeficiency 82 with systemic inflammation	AD	Heterozygous	c.259G > A (p.Asp87Asn)	VUS (PM2)
**12) A.R.Ö** **Group 3**	PROTEIN-TYROSINE KINASE SYK; *SYK*	Immunodeficiency 82 with systemic inflammation	AD	Heterozygous	c.163G > A (p.val55met)	VUS (PM2)
**13) E.U** **Group 3**	SPI1 PROTOONCOGENE; *SPI1*	Agammaglobulinemia 10	AD	Heterozygous	c.534 C > T (p.Asp178=)	VUS (PM2)

The rate of detecting a disease-causing variant by sWES was 5.45% (3/55). It seems lower than expected diagnostic yield, however the reason is this difficult group because previously no variant could be detected in these patients by tNGS and in addition they do not match any diagnostic criteria and called as unclassifed/undefined IEI patients. If we had the opportunity to study with sWES in 2017–2018 years for all 108 patients, the rate of pathogenic wariants with sWES would expected to be around 40%.

In total, tNGS + sWES allowed us to learn the genetic etiology of 40.55% of clinically diagnosed IEI patients. In another study in Turkey by Erman B et al. using only WES analysis resulted in likely genetic diagnoses for 41.1% of the patients (122 out of 297) revealing 52 novel variants [24]. In a study in South Africa, a total of 52 patients had WES only, 26 had a targeted gene panel only and two had both panel and WES. Overall, a molecular diagnosis was achieved in 30% (24/80) of patients [25].

In this cohort, familial testing allowed for two sets of parents to know their carrier and illness status. Parents of *PRKDC* patient are proven carriers of an autosomal recessive condition, the risk to have another affected child is 25% for each pregnancy. The autosomal dominant condition (*IKBKB*) in this cohort was seen not to be *de novo*, because the mother of the two affected child was highly symptomatic for severe infections and had extremely low IgG level.

In this study, the rate of familial cases is 47.1% and most of them were fathers. In our previous study, there were 37 familial cases (37/110) (33.6%) with at least one affected first-degree relative [10]. In study group, there were 6 patients with consangineous parents, one of them was with proven autosomal recessive *PRKDC* deficiency.

Familial (n = 30) and non-familial (n = 25) case groups did not show any significant difference for the percentages of detected probable disease-causing variants. This comparison has not been performed before in any studies; however, the results showed us that being a familial case did not increase the detection rate of disease-causing variants. On the other hand, we have to remember that all three disease causing variants in this study were found in familial cases group. It is obvious that we have to compare them with a larger series of familial and non-familial cases. In addition, there was no correlation between type of infections, inadequate specific antibody responses, immunoglobulin values, T-B cell numbers and detection of disease-causing variant or the efficacy of next-generation sequencing (data not shown).

Even with NGS-based approaches dramatically improving IEI diagnoses, detection rates remain below 50% illustrating the complexity and heterogeneity of these disorders. The complex mode of inheritance, limitations of the certain genomic technologies and cases caused by genes and variants not yet discovered or understood are the other reasons highlighting us the need for expanded genomic testings. The major pitfall of WES is that its coverage excludes noncoding exons such as RNA genes and intronic/intergenic regions [26, 27]. WES is presently the most cost-effective approach for IEI research and diagnostics. However, whole genome sequencing (WGS) will offer more advantages in the near future, for example in the undiagnosed cases in this study, non-coding, repetitive and structural variants might be detected by WGS. A recent study by Belkadi et al. [28] compared WES and WGS and showed that more high-quality variants were called by WGS. The cost of WGS is rapidly decreasing, and it is expected to become as affordable as WES soon, while offering the advantage of more comprehensive and accurate variant detection. 

## Supplementary Information

Below is the link to the electronic supplementary material.

Supplementary Material 1 (PDF 763 KB)

## Data Availability

Data generated in this study is either published in the article or supplementary data, or sensitive. For any inquiries, please contact corresponding author.

## References

[1] -Slade CA, Bosco JJ, Giang TB, Kruse E, Stirling RG, Cameron PU, et al. Delayed diagnosis and complications of predominantly antibody deficiencies in a cohort of Australian Adults. Front Immunol. 2018;9:694. 10.3389/fimmu.2018.00694. 29867917 10.3389/fimmu.2018.00694 PMC5960671

[2] -Seidel MG, Kindle G, Gathmann B, Quinti I, Buckland M, van Montfrans J, et al. The European Society for Immunodeficiencies (ESID) registry working definitions for the clinical diagnosis of Inborn errors of Immunity. J Allergy Clin Immunol Pract. 2019;7(6):1763–70. 10.1016/j.jaip.2019.02.004. 30776527 10.1016/j.jaip.2019.02.004

[3] -Sgrulletti M, Costagliola G, Giardino G, Graziani S, Del duca E, Cesare S, et al. The evolutionary scenario of pediatric unclassified primary antibody deficiency to adulthood. J Clin Med. 2023;12(13):4206. 10.3390/jcm12134206. 37445241 10.3390/jcm12134206 PMC10342284

[4] -Kutukculer N, Gulez N. The outcome of patients with unclassified hypogammaglobulinemia in early childhood. Pediatr Allergy Immunol. 2009;20(7):693–8. 10.1111/j.1399-3038.2008.00845.x. 19196447 10.1111/j.1399-3038.2008.00845.x

[5] -Mauracher A, Henrickson SE. Leveraging systems immunology to optimize diagnosis and treatment of inborn errors of immunity. Front Syst Biol. 2022;2:910243. 10.3389/fsysb.2022.910243. 37670772 10.3389/fsysb.2022.910243 PMC10477056

[6] -Von Hardenberg S, Klefenz I, Steinemann D, Di donato N, Baumann U, Auber B, Klemann C. Current genetic diagnostics in inborn errors of immunity. Front Pediatr. 2024;12:1279112. 10.3389/fped.2024.1279112. 38659694 10.3389/fped.2024.1279112 PMC11039790

[7] -van Dijk EL, Auger H, Jaszczyszyn Y, Thermes C. Ten years of next-generation sequencing technology. Trends Genet. 2014;30(9):418–26. 10.1016/j.tig.2014.07.001. 25108476 10.1016/j.tig.2014.07.001

[8] -Tan TY, Lunke S, Chong B, Phelan D, Fanjul-Fernandez M, Marum JE, et al. A head-to-head evaluation of the diagnostic efficacy and costs of trio versus singleton exome sequencing analysis. Eur J Hum Genet. 2019;27(12):1791–9. 10.1038/s41431-019-0471-9. 31320747 10.1038/s41431-019-0471-9 PMC6871178

[9] -Aygun A, Topyıldız E, Geyik M, Karaca NE, Durmaz A, Aksu G, Aykut A, Kutukculer N. Current genetic defects in common variable immunodeficiency patients on the geography between Europe and Asia: a single center experience. Immunol Res. 2024;72:225–33. 10.1007/s12026-023-09426-9. 37840117 10.1007/s12026-023-09426-9

[10] -Karaca NE, Severcan EU, Bilgin BG, Azarsız E, Akarcan S, Günaydın NC, Gülez N, Genel F, Aksu G, Kutukculer N. Familial inheritance and screening of first-degree relatives in common variable immunodeficiency and immunoglobulin A deficient patients. Int J Immunopathol Pharmacol. 2018;32:2058738418779458. 10.1177/2058738418779458. 29978731 10.1177/2058738418779458 PMC6073834

[11] - Kavak E, Aslan T, Karaman R, Aydin C, Ozer T, Akkoyunlu DS et al. Genomize-SEQ: An NGS data analysis platform for genomic variant classification and prioritization. 10.1101/2025.09.05.25335160

[12] -Karczewski KJ, Francioli LC, Tiao G, Cummings BB, Alföldi J, Wang Q, Mac Arthur DG. The mutational constraint spectrum quantified from variation in 141,456 humans. Nature. 2020;581:434–43. 10.1038/s41586-020-2308-7. 32461654 10.1038/s41586-020-2308-7 PMC7334197

[13] -Lek M, Karczewski KJ, Minikel EV, Samocha KE, Banks E, Fennell T. Exome Aggregation Consortium. Analysis of protein-coding genetic variation in 60,706 humans. Nature. 2016;536:285–91. 10.1038/nature19057. 27535533 10.1038/nature19057 PMC5018207

[14] - The 1000 Genomes Project Consortium. A global reference for human genetic variation. Nature. 2015;526:68–74. 10.1038/nature15393. 26432245 10.1038/nature15393 PMC4750478

[15] -Stenson PD, Mort M, Ball EV, Evans K, Hayden M, Heywood S, Hussain M, Phillips AD, Cooper DN. The Human Gene Mutation Database (HGMD^®^): optimizing its use in a clinical diagnostic or research setting. Hum Genet. 2020;139:1197–207. 10.1007/s00439-020-02199-3. 32596782 10.1007/s00439-020-02199-3 PMC7497289

[16] -Landrum MJ, Chitipiralla S, Brown GR, Chen C, Gu B, Hart J, Hoffman D, Jang W, Kaur K, Liu C, Lyoshin V, Maddipatla Z, O’Leary N, Riley G, Shi W, Zhou G, Schneider VA, Maglott DR, Holmes JB. ClinVar: improving access to variant interpretations and supporting evidence. Nucleic Acids Res. 2023;51(D1):D992–9. 10.1093/nar/gkac1011. 10.1093/nar/gkx1153 PMC5753237 29165669

[17] -Genoox. Franklin by Genoox. Available from: https://franklin.genoox.com/

[18] -Kopanos C, Tsiolkas V, Kouris A, Chapple CE, Aguilera MA, Meyer R, Massouras A. VarSome: the human genomic variant search engine. Bioinformatics 2019:35:1978–80. 10.1093/bioinformatics/bty897 10.1093/bioinformatics/bty897 PMC6546127 30376034

[19] - Richards S, Aziz N, Bale S, Bick D, Das S, Gastier-Foster J, Rehm HL. ACMG/AMP guidelines Standards and guidelines for the interpretation of sequence variants: A joint consensus recommendation of the American College of Medical Genetics and Genomics and the Association for Molecular Pathology. Genet Sci. 2015;17:405–24. 10.1038/gim.2015.30). 10.1038/gim.2015.30 PMC4544753 25741868

[20] -Aksu G, genel F, Koturoğlu G, Kurugöl Z, Kütükçüler N. Serum immunoglobulin (IgG, IgA, IgM) and IgG subclass concentrations in healthy children: a study using nephelometric technique. Turk J Pediatr. 2005;47:19–24. 16562781

[21] -Meyts I, Bosch B, Bolze A, Boisson B, Itan Y, Belkadi A, et al. Exome and genome sequencing for inborn errors of immunity. J Allergy Clin Immunol. 2016;138(4):957–69. 10.1016/j.jaci.2016.08.003. 27720020 10.1016/j.jaci.2016.08.003 PMC5074686

[22] -Rawat A, Sharma M, Vignesh P, Jindel AK, Suri D, Das J. Utility of targeted next generation sequencing for inborn errors of immunity at a tertiary care center in India. Sci Rep. 2022;12(1):10416. 10.1038/s41598-022-14522-1. 35729272 10.1038/s41598-022-14522-1 PMC9213413

[23] -Grossi A, Miano M, Lanciotti M, Fioredda F, Guardo D, Palmisani E, et al. Targeted NGS yields plentiful ultra-rare variants in inborn errors of immunity patients. Genes. 2021;12(9):1299. 10.3390/genes12091299. 34573280 10.3390/genes12091299 PMC8469131

[24] -Erman B, Aba U, Ipsir C, Pehlivan D, Aytekin C, Cildir G, et al. Genetic evaluation of the patients with clinically diagnosed inborn errors of immunity by whole exome sequencing: Results from a specialized research center for immunodeficiency in Türkiye. J Clin Immunol. 2024;44(7):157. 10.1007/s10875-024-01759-w. 38954121 10.1007/s10875-024-01759-w PMC11219406

[25] -Engelbrecht C, Urban M, Schoeman M, Paarwater B, van Coller A, Abraham DR, et al. Clinical utility of whole exome sequencing and targeted panels for identification of inborn errors of immunity in a Resource-constrained setting. Front Immunol. 2021;12665621. 10.3389/fimmu.2021.665621. 10.3389/fimmu.2021.665621 PMC8176954 34093558

[26] -Bucciol G, Van Nieuwenhove E, Moens L, Itan Y, Meyts I. Whole exome sequencing in inborn errors of immunity: use the power but mind the limits. Curr Opin Allergy Clin Immunol. 2017;17:421–30. 10.1097/ACI.0000000000000398. 28938278 10.1097/ACI.0000000000000398

[27] Lee K, Abraham RS. Next-generation sequencing for inborn errors of immunity. Hum Immunol. 2021;82:871–82. 10.1016/j.humimm.2021.02.011 10.1016/j.humimm.2021.02.011 33715910

[28] - Belkadi A, Bolze A, Itan Y, Cobat A, Vincent QB, Antipenko A, et al. Whole-genome sequencing is more powerful than whole-exome sequencing for detecting exome variants. Proc Natl Acad Sci U S A. 2015;112(17):5473–8. 10.1073/pnas.1418631112. 25827230 10.1073/pnas.1418631112 PMC4418901

