# Supplementary material for: Unclassified Inborn Errors of Immunity Patients without any Pathogenic Variant in Targeted Next-Generation Sequencing: Long-Term Follow-up and Whole Exome Sequencing Results
Source: J Clin Immunol. 2026 Mar 31;46(1):50. doi: 10.1007/s10875-026-02007-z (PMC13160999; doi:10.1007/s10875-026-02007-z)
Supplement: Supplementary file 1 — Supplementary Material 1 (PDF 763 KB) [file 10875_2026_2007_MOESM1_ESM.pdf]

The targeted next-generation sequencing analysis panel

UCSC Type	Name	Target (bp)	Missed (bp)	Covered (%)	Amplicons
Gene (CDS Only)	NSUN2	2,304	0	100	22 UCSC
Gene (CDS Only)	IL7R	1,387	0	100	12 UCSC
Gene (CDS Only)	ABCA3	5,115	0	100	47 UCSC
Gene (CDS Only)	PSMA2	705	0	100	9 UCSC
Gene (CDS Only)	ZAP70	2,021	0	100	18 UCSC
Gene (CDS Only)	CXCL10	297	0	100	4 UCSC
Gene (CDS Only)	PSTPIP1	1,251	0	100	17 UCSC
Gene (CDS Only)	SLC37A4	1,518	0	100	14 UCSC
Gene (CDS Only)	C4B	10,470	0	100	102 UCSC
Gene (CDS Only)	SLC7A7	1,536	0	100	12 UCSC
Gene (CDS Only)	IL10RA	1,737	0	100	14 UCSC
Gene (CDS Only)	SLC35C1	1,591	0	100	7 UCSC
Gene (CDS Only)	FASLG	853	0	100	10 UCSC
Gene (CDS Only)	NBN	2,265	0	100	23 UCSC
Gene (CDS Only)	CXCR4	2,130	0	100	7 UCSC
Gene (CDS Only)	NCF4	1,440	0	100	12 UCSC
Gene (CDS Only)	TNFRSF13B	882	0	100	8 UCSC
Gene (CDS Only)	RMRP	268	0	100	2 UCSC
Gene (CDS Only)	MTHFD1	2,808	0	100	28 UCSC
Gene (CDS Only)	CYBA	588	0	100	8 UCSC
Gene (CDS Only)	FRAS1	12,305	3	100	94 UCSC
Gene (CDS Only)	C1S	2,067	0	100	17 UCSC
Gene (CDS Only)	DDB1	3,423	0	100	30 UCSC
Gene (CDS Only)	C1R	1,886	0	100	16 UCSC
Gene (CDS Only)	CR2	3,279	0	100	26 UCSC
Gene (CDS Only)	CARD9	1,656	0	100	20 UCSC
Gene (CDS Only)	IGHM	1,975	0	100	11 UCSC
Gene (CDS Only)	UMPS	1,443	0	100	12 UCSC
Gene (CDS Only)	C1QC	738	0	100	5 UCSC
Gene (CDS Only)	DNAI1	2,100	0	100	21 UCSC
Gene (CDS Only)	HTR1A	1,269	0	100	8 UCSC
Gene (CDS Only)	ERMAP	1,428	0	100	13 UCSC
Gene (CDS Only)	C8A	1,755	0	100	15 UCSC
Gene (CDS Only)	STXBP2	1,964	0	100	22 UCSC
Gene (CDS Only)	CCDC103	954	0	100	5 UCSC
Gene (CDS Only)	RFX5	1,851	0	100	15 UCSC
Gene (CDS Only)	KLHDC8B	1,065	0	100	8 UCSC
Gene (CDS Only)	LYST	11,406	0	100	96 UCSC
Gene (CDS Only)	IKBKG	1,651	0	100	12 UCSC
Gene (CDS Only)	MS4A1	894	0	100	8 UCSC
Gene (CDS Only)	MYD88	1,156	0	100	8 UCSC
Gene (CDS Only)	FOXN1	1,947	0	100	14 UCSC

Gene (CDS Only)	BLM	4,254	0	100	37
Gene (CDS Only)	SDHB	843	0	100	8
Gene (CDS Only)	MBL2	747	0	100	5
Gene (CDS Only)	CAT	1,584	0	100	15
Gene (CDS Only)	SH2D1A	523	0	100	4
Gene (CDS Only)	CD59	387	0	100	4
Gene (CDS Only)	IL2RA	819	0	100	10
Gene (CDS Only)	MPO	2,238	0	100	20
Gene (CDS Only)	PLCG2	3,798	0	100	37
Gene (CDS Only)	NLRP3	3,388	0	100	20
Gene (CDS Only)	SCGB1D1	273	0	100	4
Gene (CDS Only)	VANGL1	1,702	0	100	12
Gene (CDS Only)	KIT	3,113	0	100	25
Gene (CDS Only)	LRBA	8,757	0	100	89
Gene (CDS Only)	GATA2	1,527	0	100	12
Gene (CDS Only)	PIK3R1	2,326	0	100	22
Gene (CDS Only)	FCN3	900	0	100	9
Gene (CDS Only)	GSTM1	657	0	100	9
Gene (CDS Only)	IL10	537	0	100	5
Gene (CDS Only)	PDGFRA	3,270	0	100	29
Gene (CDS Only)	ACP5	978	0	100	9
Gene (CDS Only)	TBCE	1,584	0	100	18
Gene (CDS Only)	CD8A	708	0	100	8
Gene (CDS Only)	CAPG	1,152	0	100	10
Gene (CDS Only)	ELANE	804	0	100	8
Gene (CDS Only)	STX11	864	0	100	5
Gene (CDS Only)	RAC2	579	0	100	6
Gene (CDS Only)	XIAP	1,494	0	100	13
Gene (CDS Only)	IFNGR2	1,014	0	100	9
Gene (CDS Only)	CCT5	1,626	0	100	15
Gene (CDS Only)	CCBE1	1,221	0	100	15
Gene (CDS Only)	RAB27A	666	0	100	6
Gene (CDS Only)	CFI	1,752	0	100	20
Gene (CDS Only)	CARD11	3,465	0	100	35
Gene (CDS Only)	FOXP3	1,296	0	100	13
Gene (CDS Only)	ALDH3A2	1,542	0	100	14
Gene (CDS Only)	PIGA	1,468	0	100	12
Gene (CDS Only)	TAP2	2,142	0	100	20
Gene (CDS Only)	MASP1	3,037	0	100	26
Gene (CDS Only)	PRPS1	1,049	0	100	8
Gene (CDS Only)	C8B	1,776	0	100	16
Gene (CDS Only)	LIPA	1,200	0	100	11
Gene (CDS Only)	MAP2K1	1,182	0	100	13
Gene (CDS Only)	CHST14	1,131	0	100	7
Gene (CDS Only)	ICOS	600	0	100	7

Gene (CDS Only)	IL21R	1,732	0	100	14	UCSC
Gene (CDS Only)	FCGR3A	1,168	0	100	8	UCSC
Gene (CDS Only)	HAX1	959	0	100	9	UCSC
Gene (CDS Only)	MS4A2	735	0	100	8	UCSC
Gene (CDS Only)	SF3B4	1,275	0	100	11	UCSC
Gene (CDS Only)	LAMTOR2	378	0	100	4	UCSC
Gene (CDS Only)	CD79A	867	0	100	6	UCSC
Gene (CDS Only)	CTSK	990	0	100	8	UCSC
Gene (CDS Only)	CTLA4	740	0	100	5	UCSC
Gene (CDS Only)	IL12B	987	0	100	9	UCSC
Gene (CDS Only)	WAS	1,509	0	100	15	UCSC
Gene (CDS Only)	FADD	627	0	100	4	UCSC
Gene (CDS Only)	RFXAP	819	0	100	8	UCSC
Gene (CDS Only)	PFDN4	405	0	100	7	UCSC
Gene (CDS Only)	MGP	387	0	100	5	UCSC
Gene (CDS Only)	C3	4,992	0	100	52	UCSC
Gene (CDS Only)	FAS	1,020	0	100	13	UCSC
Gene (CDS Only)	NLRP12	3,360	1	100	19	UCSC
Gene (CDS Only)	LIG4	2,736	0	100	15	UCSC
Gene (CDS Only)	PRF1	1,668	0	100	11	UCSC
Gene (CDS Only)	NFKBIA	954	0	100	10	UCSC
Gene (CDS Only)	GNPDA1	870	0	100	7	UCSC
Gene (CDS Only)	CLEC7A	854	0	100	7	UCSC
Gene (CDS Only)	IFNG	501	0	100	5	UCSC
Gene (CDS Only)	CD79B	744	0	100	8	UCSC
Gene (CDS Only)	LPIN2	2,691	0	100	24	UCSC
Gene (CDS Only)	IL17F	492	0	100	5	UCSC
Gene (CDS Only)	SEMA3E	2,424	0	100	25	UCSC
Gene (CDS Only)	C1QA	738	0	100	5	UCSC
Gene (CDS Only)	DNASE1L3	918	0	100	8	UCSC
Gene (CDS Only)	CFP	1,410	0	100	11	UCSC
Gene (CDS Only)	NOD2	3,123	0	100	24	UCSC
Gene (CDS Only)	MASP2	2,075	0	100	16	UCSC
Gene (CDS Only)	ITK	1,863	0	100	19	UCSC
Gene (CDS Only)	CD46	1,329	0	100	17	UCSC
Gene (CDS Only)	NME8	1,767	0	100	21	UCSC
Gene (CDS Only)	ITGB2	2,310	0	100	26	UCSC
Gene (CDS Only)	NCF2	1,581	0	100	18	UCSC
Gene (CDS Only)	CD3D	516	0	100	6	UCSC
Gene (CDS Only)	AICDA	597	0	100	6	UCSC
Gene (CDS Only)	PSMB8	966	0	100	10	UCSC
Gene (CDS Only)	HPGD	909	0	100	10	UCSC
Gene (CDS Only)	NHEJ1	900	0	100	8	UCSC
Gene (CDS Only)	DNMT3B	2,740	0	100	25	UCSC
Gene (CDS Only)	GPI	1,916	0	100	22	UCSC

Gene (CDS Only)	TERC	451	0	100	3 UCSC
Gene (CDS Only)	RAG2	1,584	0	100	8 UCSC
Gene (CDS Only)	STIM1	2,525	0	100	20 UCSC
Gene (CDS Only)	BCL10	702	0	100	7 UCSC
Gene (CDS Only)	RAG1	3,132	0	100	16 UCSC
Gene (CDS Only)	AK2	992	0	100	10 UCSC
Gene (CDS Only)	LRRC8A	2,433	0	100	14 UCSC
Gene (CDS Only)	FKBP14	636	0	100	7 UCSC
Gene (CDS Only)	MTAP	852	0	100	9 UCSC
Gene (CDS Only)	IRAK4	1,501	0	100	15 UCSC
Gene (CDS Only)	CD19	1,767	0	100	17 UCSC
Gene (CDS Only)	COX4I2	516	0	100	5 UCSC
Gene (CDS Only)	MLPH	1,803	0	100	18 UCSC
Gene (CDS Only)	TCN2	1,380	0	100	12 UCSC
Gene (CDS Only)	IL10RB	985	0	100	8 UCSC
Gene (CDS Only)	FCGR3B	1,115	0	100	7 UCSC
Gene (CDS Only)	STK4	1,464	0	100	12 UCSC
Gene (CDS Only)	C7	2,532	0	100	24 UCSC
Gene (CDS Only)	TAZ	879	0	100	15 UCSC
Gene (CDS Only)	MAN2B1	3,116	0	100	28 UCSC
Gene (CDS Only)	CYBB	1,762	0	100	20 UCSC
Gene (CDS Only)	TNFRSF13C	555	0	100	5 UCSC
Gene (CDS Only)	KRAS	687	0	100	7 UCSC
Gene (CDS Only)	MBTPS2	1,560	0	100	18 UCSC
Gene (CDS Only)	MVK	1,191	0	100	11 UCSC
Gene (CDS Only)	C2	2,332	0	100	23 UCSC
Gene (CDS Only)	CD3E	624	0	100	9 UCSC
Gene (CDS Only)	C4A	10,750	0	100	102 UCSC
Gene (CDS Only)	SKIV2L	3,741	0	100	36 UCSC
Gene (CDS Only)	USB1	910	0	100	9 UCSC
Gene (CDS Only)	G6PC3	1,041	0	100	10 UCSC
Gene (CDS Only)	SFTPB	1,182	0	100	12 UCSC
Gene (CDS Only)	CIITA	3,393	0	100	30 UCSC
Gene (CDS Only)	TCIRG1	2,558	0	100	25 UCSC
Gene (CDS Only)	SMARCAL1	2,865	0	100	24 UCSC
Gene (CDS Only)	CD247	528	0	100	8 UCSC
Gene (CDS Only)	FUCA1	1,401	0	100	12 UCSC
Gene (CDS Only)	AGA	1,195	0	100	12 UCSC
Gene (CDS Only)	RFXANK	864	0	100	8 UCSC
Gene (CDS Only)	CD40LG	793	0	100	7 UCSC
Gene (CDS Only)	SLC17A5	1,488	0	100	18 UCSC
Gene (CDS Only)	NEU1	1,248	0	100	10 UCSC
Gene (CDS Only)	STAT1	2,333	0	100	26 UCSC
Gene (CDS Only)	C1QB	762	0	100	5 UCSC
Gene (CDS Only)	CFD	762	0	100	10 UCSC

Gene (CDS Only)	UNG	1,254	0	100	9	UCSC
Gene (CDS Only)	TAPBP	1,562	0	100	14	UCSC
Gene (CDS Only)	COLEC11	1,161	0	100	12	UCSC
Gene (CDS Only)	NRAS	570	0	100	5	UCSC
Gene (CDS Only)	MPV17	531	0	100	7	UCSC
Gene (CDS Only)	C6	2,805	0	100	25	UCSC
Gene (CDS Only)	IGLL1	691	0	100	5	UCSC
Gene (CDS Only)	ZBTB24	3,096	0	100	14	UCSC
Gene (CDS Only)	GFI1	1,269	0	100	11	UCSC
Gene (CDS Only)	C9	1,680	0	100	16	UCSC
Gene (CDS Only)	BUB1B	3,153	0	100	33	UCSC
Gene (CDS Only)	MRO	888	0	100	8	UCSC
Gene (CDS Only)	PNP	870	0	100	9	UCSC
Gene (CDS Only)	MAGT1	1,104	0	100	14	UCSC
Gene (CDS Only)	IL1RN	673	0	100	8	UCSC
Gene (CDS Only)	SPINK5	3,384	0	100	38	UCSC
Gene (CDS Only)	CD3G	549	0	100	7	UCSC
Gene (CDS Only)	IL2RG	1,117	0	100	11	UCSC
Gene (CDS Only)	POLE	6,861	0	100	64	UCSC
Gene (CDS Only)	POLH	2,142	0	100	18	UCSC
Gene (CDS Only)	BTK	2,015	0	100	24	UCSC
Gene (CDS Only)	LRRC6	1,401	2	99.9	17	UCSC
Gene (CDS Only)	SLC29A3	1,595	1	99.9	11	UCSC
Gene (CDS Only)	LRIG1	3,282	3	99.9	26	UCSC
Gene (CDS Only)	CASP8	2,131	3	99.9	13	UCSC
Gene (CDS Only)	LIG1	2,760	4	99.9	30	UCSC
Gene (CDS Only)	SEC14L3	1,263	3	99.8	14	UCSC
Gene (CDS Only)	C5	5,031	9	99.8	57	UCSC
Gene (CDS Only)	MAP2K2	1,203	3	99.8	14	UCSC
Gene (CDS Only)	MYO5A	5,568	9	99.8	54	UCSC
Gene (CDS Only)	TNFSF11	954	2	99.8	8	UCSC
Gene (CDS Only)	DOCK8	6,428	15	99.8	56	UCSC
Gene (CDS Only)	ATM	9,206	21	99.8	105	UCSC
Gene (CDS Only)	TMC6	2,418	6	99.8	26	UCSC
Gene (CDS Only)	GLB1	2,189	4	99.8	18	UCSC
Gene (CDS Only)	BRAF	2,301	5	99.8	25	UCSC
Gene (CDS Only)	IRF8	1,281	4	99.7	10	UCSC
Gene (CDS Only)	EPG5	7,740	20	99.7	65	UCSC
Gene (CDS Only)	CFH	3,717	14	99.6	41	UCSC
Gene (CDS Only)	VPS13B	12,524	56	99.6	110	UCSC
Gene (CDS Only)	SP110	2,367	11	99.5	23	UCSC
Gene (CDS Only)	FREM2	9,510	44	99.5	66	UCSC
Gene (CDS Only)	IFNGR1	1,470	9	99.4	13	UCSC
Gene (CDS Only)	CASP1	1,266	7	99.4	10	UCSC
Gene (CDS Only)	HEATR2	2,568	16	99.4	20	UCSC

Gene (CDS Only)	IL17RA	2,601	16	99.4	23	UCSC
Gene (CDS Only)	AIRE	1,638	11	99.3	21	UCSC
Gene (CDS Only)	FASN	7,536	58	99.2	68	UCSC
Gene (CDS Only)	ORAI1	912	7	99.2	6	UCSC
Gene (CDS Only)	TBX1	1,786	15	99.2	18	UCSC
Gene (CDS Only)	WNT10A	1,254	10	99.2	9	UCSC
Gene (CDS Only)	FLG	12,186	106	99.1	66	UCSC
Gene (CDS Only)	CCDC114	2,013	18	99.1	19	UCSC
Gene (CDS Only)	BLNK	1,528	15	99	18	UCSC
Gene (CDS Only)	DNAH9	13,509	137	99	104	UCSC
Gene (CDS Only)	POLQ	7,773	76	99	63	UCSC
Gene (CDS Only)	CFHR1	993	11	98.9	10	UCSC
Gene (CDS Only)	CASP10	1,821	22	98.8	16	UCSC
Gene (CDS Only)	XPC	2,949	41	98.6	23	UCSC
Gene (CDS Only)	MEFV	2,565	39	98.5	21	UCSC
Gene (CDS Only)	TMC8	2,181	34	98.4	21	UCSC
Gene (CDS Only)	ITPKC	2,052	35	98.3	15	UCSC
Gene (CDS Only)	ARHGAP4	2,961	53	98.2	31	UCSC
Gene (CDS Only)	SDHC	722	14	98.1	7	UCSC
Gene (CDS Only)	IGHG2	1,132	21	98.1	8	UCSC
Gene (CDS Only)	TAP1	2,427	48	98	18	UCSC
Gene (CDS Only)	JAK3	3,375	87	97.4	30	UCSC
Gene (CDS Only)	STAT5B	2,364	62	97.4	20	UCSC
Gene (CDS Only)	TNFRSF1A	1,368	38	97.2	18	UCSC
Gene (CDS Only)	UNC93B1	1,793	51	97.2	16	UCSC
Gene (CDS Only)	DCLRE1C	2,105	60	97.1	19	UCSC
Gene (CDS Only)	ADA	1,092	33	97	13	UCSC
Gene (CDS Only)	STAT3	2,548	84	96.7	26	UCSC
Gene (CDS Only)	PGM3	2,429	80	96.7	20	UCSC
Gene (CDS Only)	TYK2	3,564	128	96.4	31	UCSC
Gene (CDS Only)	CD81	711	26	96.3	11	UCSC
Gene (CDS Only)	NLRP1	4,574	175	96.2	34	UCSC
Gene (CDS Only)	HYDIN	16,139	607	96.2	120	UCSC
Gene (CDS Only)	LMNB2	1,863	72	96.1	20	UCSC
Gene (CDS Only)	SLC39A4	2,343	94	96	20	UCSC
Gene (CDS Only)	CD40	862	36	95.8	11	UCSC
Gene (CDS Only)	IL12RB1	2,114	91	95.7	22	UCSC
Gene (CDS Only)	RECQL4	3,627	159	95.6	32	UCSC
Gene (CDS Only)	DNAL1	608	29	95.2	8	UCSC
Gene (CDS Only)	FERMT3	2,129	103	95.2	21	UCSC
Gene (CDS Only)	PTPRC	4,085	196	95.2	43	UCSC
Gene (CDS Only)	ITCH	2,719	134	95.1	27	UCSC
Gene (CDS Only)	NCF1	1,173	128	89.1	11	UCSC
Gene (CDS Only)	RPGR	4,154	996	76	31	UCSC
